# Papain-like cysteine proteases in *Carica papaya*: lineage-specific gene duplication and expansion

**DOI:** 10.1186/s12864-017-4394-y

**Published:** 2018-01-06

**Authors:** Juan Liu, Anupma Sharma, Marie Jamille Niewiara, Ratnesh Singh, Ray Ming, Qingyi Yu

**Affiliations:** 1 FAFU and UIUC-SIB Joint Center for Genomics and Biotechnology; Fujian Provincial Key Laboratory of Haixia Applied Plant Systems Biology; Key Laboratory of Genetics, Breeding and Multiple Utilization of Corps, Ministry of Education; College of Life Science; Fujian Agriculture and Forestry University, Fuzhou, 350002 Fujian China; 2 Texas A&M AgriLife Research Center at Dallas, Texas A&M University System, Dallas, TX 75252 USA; 3 Department of Plant Biology, School of Integrative Biology, University of Illinois at Urbana-Champaign, Urbana, IL 61801 USA; 4 Department of Plant Pathology & Microbiology, Texas A&M University, College Station, TX 77843 USA

**Keywords:** *Carica papaya*, Papain-like cysteine proteases, Papaya latex, Gene duplication

## Abstract

**Background:**

Papain-like cysteine proteases (PLCPs), a large group of cysteine proteases structurally related to papain, play important roles in plant development, senescence, and defense responses. Papain, the first cysteine protease whose structure was determined by X-ray crystallography, plays a crucial role in protecting papaya from herbivorous insects. Except the four major PLCPs purified and characterized in papaya latex, the rest of the PLCPs in papaya genome are largely unknown.

**Results:**

We identified 33 PLCP genes in papaya genome. Phylogenetic analysis clearly separated plant PLCP genes into nine subfamilies. PLCP genes are not equally distributed among the nine subfamilies and the number of PLCPs in each subfamily does not increase or decrease proportionally among the seven selected plant species. Papaya showed clear lineage-specific gene expansion in the subfamily III. Interestingly, all four major PLCPs purified from papaya latex, including papain, chymopapain, glycyl endopeptidase and caricain, were grouped into the lineage-specific expansion branch in the subfamily III. Mapping PLCP genes on chromosomes of five plant species revealed that lineage-specific expansions of PLCP genes were mostly derived from tandem duplications. We estimated divergence time of papaya PLCP genes of subfamily III. The major duplication events leading to lineage-specific expansion of papaya PLCP genes in subfamily III were estimated at 48 MYA, 34 MYA, and 16 MYA. The gene expression patterns of the papaya PLCP genes in different tissues were assessed by transcriptome sequencing and qRT-PCR. Most of the papaya PLCP genes of subfamily III expressed at high levels in leaf and green fruit tissues.

**Conclusions:**

Tandem duplications played the dominant role in affecting copy number of PLCPs in plants. Significant variations in size of the PLCP subfamilies among species may reflect genetic adaptation of plant species to different environments. The lineage-specific expansion of papaya PLCPs of subfamily III might have been promoted by the continuous reciprocal selective effects of herbivore attack and plant defense.

**Electronic supplementary material:**

The online version of this article (10.1186/s12864-017-4394-y) contains supplementary material, which is available to authorized users.

## Background

Papain-like cysteine proteases (PLCPs), belonging to protease family C1A of clan CA, are the most abundant among the cysteine proteases [1]. PLCPs are found in a wide range of living organisms, including virus [2], bacteria [3], yeast [4], protozoa, plants, and animals [1, 5]. The origin of the PLCP family likely occurred prior to the divergence of the principal eukaryotic lineages [6, 7].

Papain is the first cysteine protease isolated and characterized from *Carica papaya* [8] and it is also the first cysteine protease whose structure was determined by X-ray crystallography [9]. PLCPs are structurally related to papain and characterized by a typical papain fold, which consists of two sequentially connected domains: an α-helix and a β-sheet domain [9]. The active-site cleft, containing the catalytic triad Cys-His-Asn, forms at the two-domain interface [9]. Given their high destructive potential, the activity of PLCPs is tightly regulated. Like other proteolytic enzymes, PLCPs are synthesized as inactive precursors which contain an autoinhibitory prodomain to prevent unwanted protein degradation [10]. The prodomain can block access of substrate to the active site, and also plays roles in protein folding and subcellular targeting [11, 12]. The activity of PLCPs also depends on pH and the presence of their endogenous inhibitors or activators [13]. There is a fine balance between PLCPs and their endogenous inhibitors to help control the activation and catabolism of many PLCPs.

PLCPs are involved in diverse biological processes, including senescence [14] and defense responses [15, 16]. As an essential part of the proteolytic machinery, PLCPs are responsible for intracellular protein degradation and are key enzymes in the regulation of programmed cell death (PCD). Cell death is a tightly regulated biological process that functions in many aspects of plant development and in the responses to biotic and abiotic stresses. Increased activity of PLCPs was observed in developing and germinating seeds [17], fruits [18] and senescing organs [19, 20]. PLCPs also play essential roles in plant-pathogen/pest interactions. Activity of PLCPs is required to trigger plant immune responses and fulfill effective defense against pathogen infection [15, 21, 22]. Meanwhile, PLCPs are often targeted by pathogen-derived effectors to suppress plant immune responses [23–27]. Therefore, the continuous co-evolutionary arms race between pathogens and their hosts might have driven a more rapid evolution of plant PLCPs compared to the rest of plant genomes. In addition, PLCPs are also tightly linked to resistance to herbivore attack. Papain, one of the PLCPs in latex exuding from wounds, plays a crucial role in protecting papaya from herbivorous insects, such as lepidopteran larvae [28]. Similarly, a 33-kDa PLCP in maize confers resistance to caterpillars by damaging their digestive systems [29, 30].

Plant PLCPs were grouped into nine subfamilies primarily based on their structural characteristics [31]. Genes belonging to large families may have evolved through tandem duplications, genome-wide duplications, or large-scale segmental duplications. And gene duplicates provide an essential source of genetic raw material for evolutionary novelty. Therefore, understanding the evolutionary relationships between PLCPs will help identify the function of individual PLCP.

*Carica papaya* represents a special system to study PLCPs. It is one of the plant species that can exude latex upon tissue damage. Papaya latex is rich in PLCPs, which are responsible for the defensive activities [28]. Four major PLCPs, papain, chymopapain, glycyl endopeptidase and caricain, have been purified and characterized in papaya latex [32]. In this study, we performed genome-wide identification of PLCPs in papaya genome, analyzed the expression patterns for each PLCP, and refined the evolutionary relationships of PLCPs from major monocot and dicot plant species. Our data demonstrated that the major PLCP components of papaya latex had evolved from lineage-specific expansion of PLCPs in subfamily III via stepwise duplication events. The lineage-specific expansion of papaya PLCPs of subfamily III might have been promoted by the continuous reciprocal selective effects of herbivore attack and plant defense.

## Methods

### Plant materials

Papaya variety Zhonghuang were grown and maintained at a greenhouse in Fujian Agriculture and Forest University. Leaf tissue, male flowers at 5 mm and 35 mm long, and green and 50% yellow fruits were collected. The fruit skin and flesh were manually dissected before RNA extraction. Detailed information of developing stages and sex types for each sample is given in Additional file 1. The harvested tissues were snap-frozen by dropping directly into liquid nitrogen and stored in a freezer at -80 °C until RNA extraction.

### RNA isolation

Plant tissues were ground in liquid nitrogen to a fine powder using a mortar and then used for total RNA extraction using Qiagen RNeasy Plant Mini Kit (Qiagen) following the manufacturer’s protocol. DNA contamination was eliminated using Ambion DNA-free DNA Removal Kit (Life Technologies).

### Identification of papain-like cysteine proteases in selected genomes

All gene models used in this study were downloaded from Phytozome v11 (https://phytozome.jgi.doe.gov/pz/portal.html). Initial identification of PLCPs was carried out using HMMER v3.1 [33] against the Pfam Peptidase_C1 domain (PF00112) (http://pfam.xfam.org/) with default settings. The identified PLCPs were then used as queries to search against the NCBI Conserved Domains Database (CDD) and against the NCBI protein database to confirm that they contain the Peptidase_C1 domain and have homology to the PLCP family members.

### Phylogenetic analysis

Full-length amino acid sequences of PLCPs were used for initial multiple sequence alignment by MUSCLE v3.8.31 [34] with default parameters. Manual correction was done to remove poorly aligned regions using BioEdit v7.2.0 [35]. The resulting alignments were used to construct the phylogenetic trees by PhyML v3.0 with Smart Model Selection [36]. The output trees were further edited using MEGA 5.0 [37].

### Gene expression analysis of papaya PLCPs in five papaya tissues at different developing stages

The raw RNA-Seq reads were downloaded from NCBI (Additional file 2) and trimmed with TRIMMOMATIC v0.30 to remove Illumina adapter sequences, any base below quality phred score 3 and any read less than 36 bp in length [38]. The trimmed sequence reads were aligned to repeat-masked papaya genome [39] using TopHat (v2.1.1) with default settings [40]. The uniquely mapped reads were then used to calculate the number of reads falling into each gene and normalized to fragments per kilobase of exon per million fragments mapped (FPKM) using Cufflinks (v2.2.1) followed by Cuffnorm (v2.2.1) with default settings and papaya gene model annotation provided. The normalized FPKM values of papaya PLCPs were log2 transformed and used to compute the hierarchical clustering using the online software MeV (using the Pearson correlation coefficient and average linkage).

### Quantitative real-time PCR (qPCR)

The primers used for quantitative real-time PCR were designed using Primer Premier 5 software (http://www.premierbiosoft.com/primerdesign/). Ubiquitin gene was included as an internal reference gene for normalization as suggested by Zhu et al. [41]. Primer sequences are listed in Additional file 3. Total RNA was extracted from different papaya tissues with TRIzol reagent using the method as described by Lin et al. [42]. Approximately 1 μg of total RNA was used as template for reverse transcription using the PrimeScript 1st strand cDNA synthesis kit (TaKaRa). The synthesized 1st strand cDNA was then diluted 10-fold and 1 μl of diluted cDNA was used in the qPCR reaction. The qPCR was performed in a 15 μl reaction containing 1.0 μl of cDNA template, 1.5 μl of 2 μM forward and reverse primer mix, 7.5 μl of Perfecta SYBR Green FastMix (Quanta BioSciences), and 5.0 μl of Ambion nuclease-free water. The reaction was run on a CFX96 Real-Time PCR Detection System (Bio-Rad). The PCR program was as follows: 95 °C for 3 min, 45 cycles of: 95 °C for 10 S, 60 °C for 30 S, 95 °C for 10S, followed by melt curve. Three technical replicates for each sample were performed for qPCR analysis. The 2-^△△Ct^ method was used for relative gene expression analysis. Analysis of variance (ANOVA) was used to test the significance of expression level.

### Conserved domain identification, gene duplication analysis, and chromosomal distribution of PLCPs

Conserved Domains Database (CDD) from NCBI was used to identify conserved domains in PLCPs. The ‘duplicate_gene_classifier’ program of the MCScanX package [43] was used to classify the gene duplication events of PLCPs in the seven selected plant species. We used an in-house python script to draw chromosomal distribution of PLCPs in five plant species.

### Estimation of divergence times

Exon and intron regions of gene pairs were manually aligned using BioEdit [35]. DnaSP v5.0 [44] was used to calculate synonymous substitutions per synonymous site (*Ks*), nonsynonymous substitutions per nonsynonymous site (*Ka*), and synonymous and noncoding (silent) substitutions per silent site (*Ksil*). Divergence times were determined using *Ksil* and the methods described by Li [45] using a mean substitution rate of 7.1 × 10^−9^ substitutions/site/year estimated in *A. thaliana* based on mutation accumulation experiments [46], corrected by a factor of 0.672 for papaya as described by VanBuren et al. [47].

## Results

### Identification and phylogenetic analysis of papain-like cysteine proteases

By searching for the Peptidase_C1 domain, we identified 33 PLCP genes in papaya genome. We double-checked the Peptidase_C1 domain in these 33 genes using the NCBI Conserved Domain Database and all of them contain a complete Peptidase_C1 domain. We further blasted search the protein sequences of these genes into GenBank and all of them had the closest homologs belonging to PLCP family. We therefore finalized the list of papaya PLCP genes (Table 1).

**Table 1:** List of papain-like cysteine protease genes identified in papaya genome

Gene name	Subfamily	Length(aa)	Gene Model ID
*CpRD21A*	I	471	evm.TU.supercontig_200.16
*CpRD21B*	I	353	evm.TU.supercontig_21.155
*CpRD21C*	I	385	evm.TU.supercontig_55.99
*CpCEP1*	II	358	evm.TU.supercontig_33.69
*CpCEP2*	II	362	evm.TU.supercontig_4.30
*CpXCP5* (*Papain*)	III	284	evm.TU.supercontig_1120.1
*CpXCP6*	III	237	evm.TU.supercontig_155.5
*CpXCP7* (*CpGlycyl endopeptidase*)	III	348	evm.TU.supercontig_155.6
*CpXCP1*	III	348	evm.TU.supercontig_232.3
*CpXCP3*	III	361	evm.TU.contig_27841.1
*CpXCP2*	III	357	evm.TU.supercontig_286.15
*CpXCP8* (*CpChymopapain*)	III	361	evm.TU.supercontig_286.8
*CpXCP4*	III	345	evm.TU.contig_32339.1
*CpXCP9*	III	336	evm.TU.supercontig_547.1
*CpXBCP1*	IV	130	evm.TU.supercontig_698.3
*CpXBCP2*	IV	421	evm.TU.supercontig_698.4
*CpXBCP3*	IV	488	evm.TU.supercontig_7.74
*CpTHI1*	V	349	evm.TU.supercontig_209.8
*CpPAP2*	VI	337	evm.TU.supercontig_103.39
*CpPAP3*	VI	337	evm.TU.supercontig_103.40
*CpPAP4*	VI	337	evm.TU.supercontig_103.41
*CpPAP5*	VI	339	evm.TU.supercontig_209.15
*CpPAP6*	VI	310	evm.TU.supercontig_209.18
*CpPAP1*	VI	42	evm.TU.contig_45135.1
*CpPAP7*	VI	334	evm.TU.supercontig_7.54
*CpPAP8*	VI	334	evm.TU.supercontig_7.56
*CpPAP9*	VI	325	evm.TU.supercontig_899.2
*CpPAP10*	VI	217	evm.TU.supercontig_899.3
*CpRD19A*	VII	203	evm.TU.supercontig_150.50
*CpRD19B*	VII	362	evm.TU.supercontig_3257.1
*CpRD19C*	VII	366	evm.TU.supercontig_53.142
*CpAALP*	VIII	355	evm.TU.supercontig_28.27
*CpCTB1*	IX	347	evm.TU.supercontig_57.54

To study the evolutionary relationships among the newly identified PLCPs, we selected six additional plant species for phylogenetic analysis. These six species include three dicots (*Arabidopsis thaliana*, *Vitis vinifera*, and *Populus trichocarpa*), two monocots (*Oryza sativa* and *Sorghum bicolor*), and one basal angiosperm (*Amborella trichopoda*). Using the same method described above, we identified 27 PLCP genes in *A. trichopoda*, 49 in *P. trichocarpa*, 32 in *A. thaliana*, 24 in *V. vinifera*, 45 in *S. bicolor*, and 45 in *O. sativa* (Table 2).

**Table 2:** Distribution of papain-like cysteine proteases over subfamilies in seven selected plant species

Species	PLCP Subfamily									Total
	I	II	III	IV	V	VI	VII	VIII	IX	
*Carica papaya*	3	2	9	3	1	10	3	1	1	33
*Arabidopsis thaliana*	9	3	2	1	1	7	4	2	3	32
*Populus trichocarpa*	5	6	4	5	1	19	5	1	3	49
*Vitis vinifera*	3	2	2	2	1	7	5	1	1	24
*Oryza sativa*	3	12	3	1	12	9	3	1	1	45
*Sorghum bicolor*	7	10	3	1	4	15	3	1	1	45
*Amborella trichopoda*	2	1	2	2	0	16	2	1	1	27
Total	32	36	25	15	20	83	25	8	11	255

**Fig. 1:**
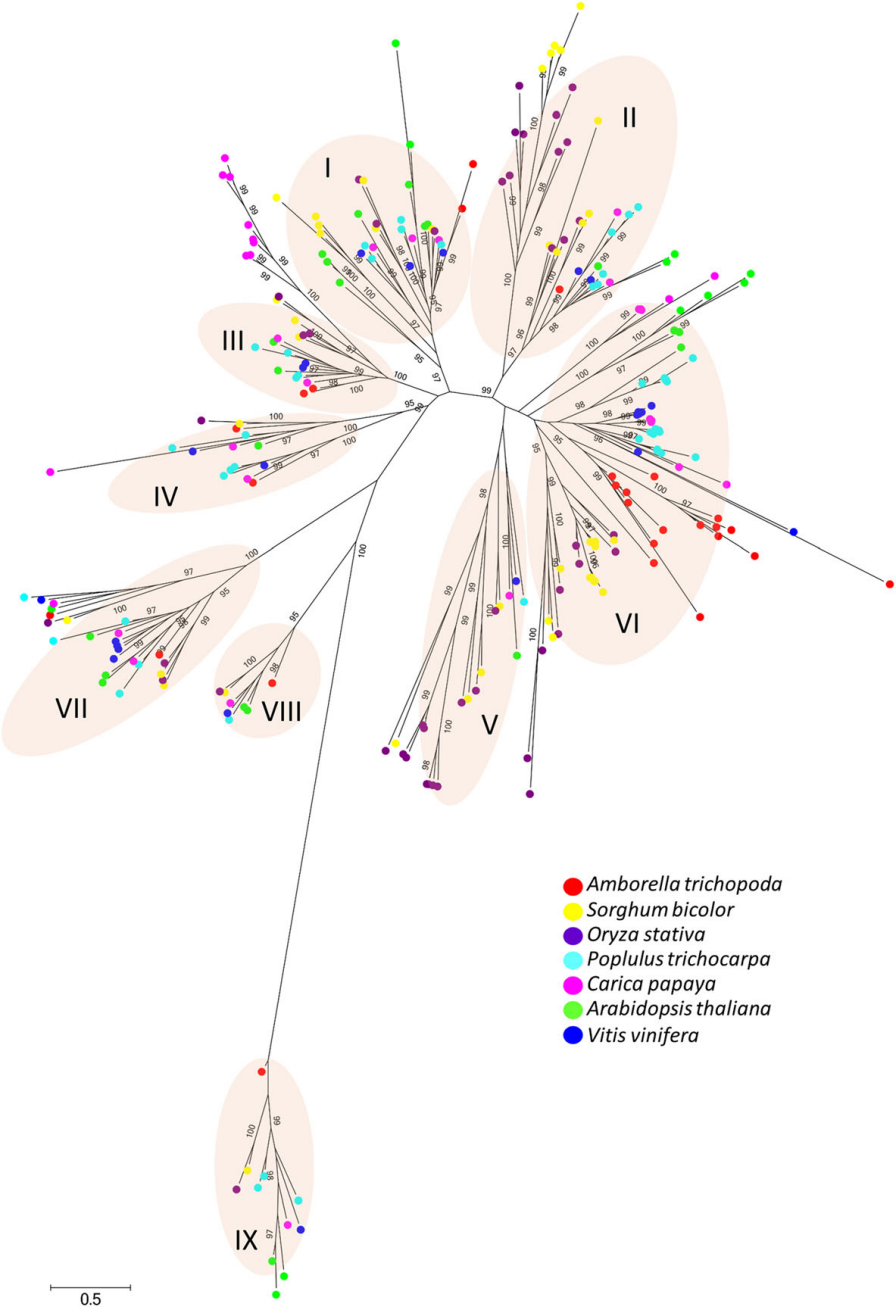
The Phylogenetic tree of 255 PLCP genes identified in papaya, *Amborella*, *Populus*, *Arabidopsis*, grape, sorghum and rice genomes. The phylogenetic analysis grouped the 255 PLCP genes into nine PLCP subfamilies. Bootstrap values ≥95 were shown

A phylogenetic tree was built using PLCP genes identified from the seven representative plant species (Fig. 1). The phylogenetic tree separated PLCP genes into nine different subfamilies, which is consistent with previous study by Richau et al. [31]. Based on the phylogenetic analysis, we named the 33 papaya PLCP genes to keep the same naming system as reported by Richau et al. [31] (Table 1). Subfamilies I-VI, containing cathepsin L-like PLCPs, are relatively related to each other. Subfamilies VII, VIII and IX contain cathepesin F-like, cathepsin H-like and cathepsin B-like PLCPs, respectively, and they are distinct from subfamilies I-VI.

PLCP genes of the seven plant species are not equally distributed among the nine subfamilies (Table 2). Subfamily VI contains the largest number of PLCP genes among the nine subfamilies, while subfamily VIII contains the least number of PLCP genes, about one-tenth of the total number of PLCP genes in subfamily VI. The number of PLCPs in each subfamily does not proportionally increase or decrease among the seven plant species (Fig. 1, Table 2). Our phylogenetic analysis revealed extensive lineage-specific gene expansion. A total of 25 PLCPs from the seven plant species were grouped into the subfamily III and nine of them are from papaya. Papaya showed clear lineage-specific gene expansion in the subfamily III. And all four major PLCPs purified from papaya latex, including papain, were grouped into the lineage-specific expansion branch in the subfamily III. The two monocot species, rice and sorghum, displayed distinct lineage-specific gene expansion in the subfamily II. Rice showed significant expansion in the subfamily V compared with the rest of the plant species. The basal angiosperm, *A. trichopoda*, showed clear lineage-specific gene expansion in the subfamily VI. The total number of *A. trichopoda* PLCP genes in the subfamily VI accounts for more than 50% of the total number of PLCP genes in *A. trichopoda* genome.

### Lineage-specific expansion of PLCP genes of subfamily III in papaya

Papaya displays clear lineage-specific gene expansion in subfamily III and all PLCPs purified from papaya latex were grouped into this lineage-specific expansion branch. We therefore built a separate phylogenetic tree only for subfamily III PLCP genes using PLCP genes from 53 plant species whose genome sequences were available in Phytozome v11.

**Fig. 2:**
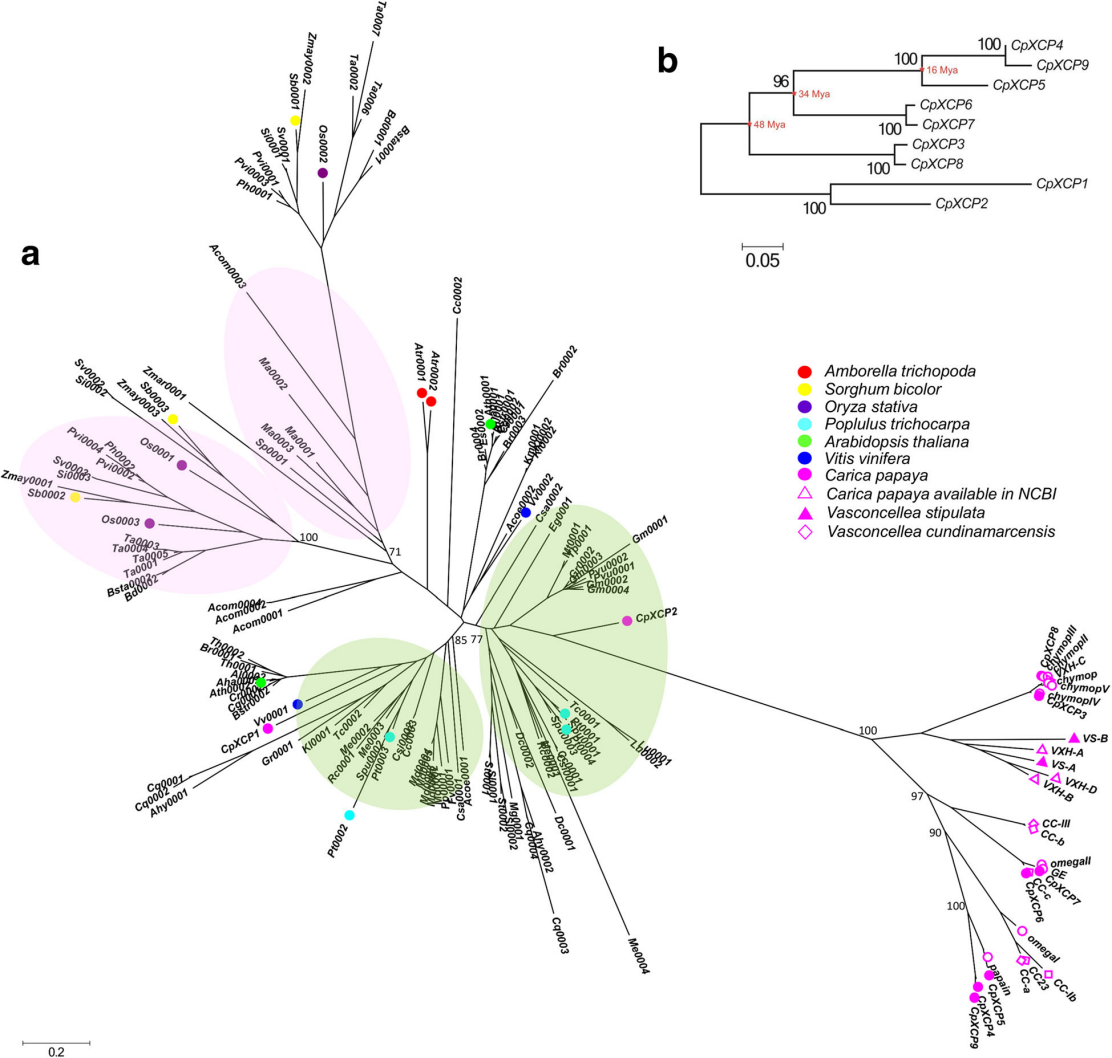
**a** The phylogenetic tree of 155 PLCP genes of subfamily III identified in 53 plant species whose genome sequences were available in Phytozome database and papaya relative species available in Genbank. The pink color highlights the major subclades of the monocot group and the green color highlights the major subclades of the dicot group. Bootstrap values of major branches were shown. **b** Phylogenetic relations of the nine PLCP genes of subfamily III in papaya using the Neighbor-Joining method with 1000 replicates in the bootstrap test. Estimated ages of major duplication events leading to lineage-specific expansion of papaya PLCP genes in subfamily III were shown

Using the same method and criteria described in the previous section, we identified 134 PLCP genes of subfamily III from 53 plant species (Additional file 4). We also downloaded and included PLCP genes of *C. papaya* and its relative *Vasconcellea* species that were available in GenBank in our phylogenetic analysis. The whole list of PLCP genes of subfamily III used for phylogenetic analysis is given in Additional file 5. Phylogenetic analysis grouped the 155 PLCP genes of subfamily III into two major clades, one containing all the PLCP genes from monocots and the other one containing all the PLCP genes from dicots (Fig. 2). Each clade was further divided into two subclades, suggesting PLCP genes of subfamily III had undergone independent duplication events after the divergence of monocots and dicots from a common ancestor. Except papaya, all the other plant species didn’t show clear lineage-specific gene expansion in this subfamily. Among the nine papaya PLCP genes of subfamily III identified in this study, eight of them were closely clustered and were derived from lineage-specific gene expansion. Interestingly, all the four major PLCPs purified from papaya latex, papain, chymopapain, glycyl endopeptidase and caricain, are included in this cluster. All the latex PLCP genes of *C. papaya* and *Vasconcellea* species downloaded from GenBank were also grouped into the lineage-specific expansion branch. Our phylogenetic analysis showed that the lineage-specific gene expansion initiated before *Carica* and *Vasconcellea* diverged from a common ancestor and had undergone additional expansion after these two genera split from a common ancestor.

### Estimating the ages of gene duplication events of subfamily III PLCP genes in papaya

We selected paralogous gene pairs of subfamily III PLCP genes in papaya based on their phylogenetic relationships and analyzed their sequence divergence to trace evolutionary history of the lineage-specific gene duplication events in papaya. We calculated synonymous (*K*_*s*_) and non-synonymous (*K*_*a*_) divergence between each paralogous gene pair and assessed the ratio of non-synonymous to synonymous divergence in order to infer the degree of functional constraint acting on the duplicated genes. In general, the *K*_*a*_/*K*_*s*_ ratio is greater than 1 when fixation of nonsynonymous substitution is faster than that of synonymous substitutions, which means that positive selection fixes amino acid changes faster than silent ones [48]. Conversely, when deleterious substitutions are eliminated by purifying selection (negative selection), the *K*_*a*_/*K*_*s*_ ratio is less than 1. The *K*_*a*_/*K*_*s*_ ratio is close to 1 when the positive and negative selection forces balance each other. The total number of synonymous and non-synonymous sites and degree of divergence between each paralogous gene pair are summarized in Additional file 6. All gene pairs except one have *K*_*a*_/*K*_*s*_ ratios that are less than 1, suggesting divergence of these duplicated paralogous gene pairs had been functionally constrained. Since the *K*_*a*_/*K*_*s*_ ratio of the gene pair *CpXCP3*/*CpXCP8* is only slightly greater than 1, it is difficult to determine whether this duplicated paralogous gene pair had been under positive selection.

We also assessed the degree of silent site nucleotide divergence (*K*_*sil*_) between the duplicated paralogous gene pairs (Additional files 7 and 8). Overall, the degree of silent site divergence matched the phylogenetic distance between the paralogous gene pairs. The gene pairs having long phylogenetic distance showed the high degree of silent site divergence. Using a molecular clock of 7.1 × 10^−9^ synonymous substitutions per site per year [46], corrected by a factor of 0.672 [47], we estimated the ages of gene duplication events of subfamily III PLCPs in papaya (Fig. 2b, Additional files 7 and 8). The major duplication events leading to lineage-specific expansion of papaya PLCP genes in subfamily III were estimated at 48 MYA, 34 MYA, and 16 MYA (Fig. 2b, Additional file 7).

### Gene expression profile of the papaya PLCP genes in different tissues

**Fig. 3:**
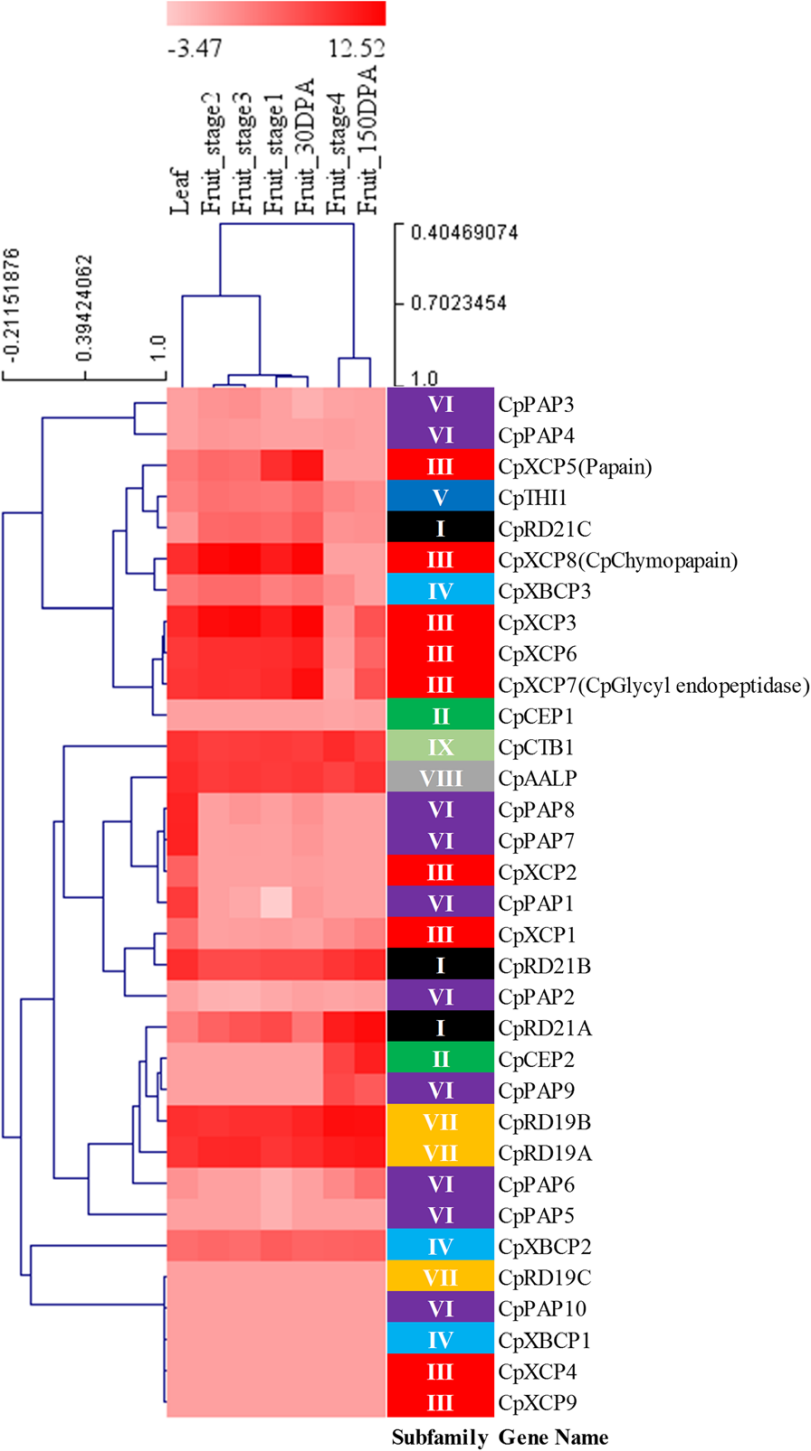
Hierarchical clustering of expression profiles of papaya PLCP genes across different tissues

Papaya latex is usually harvested from immature green papaya fruits. To study the expression pattern of the papaya PLCP genes, we obtained normalized FPKM values of papaya PLCP genes based on RNA-Seq libraries prepared from leaf tissue and fruits at six different developmental stages. The expression profiles of the papaya PLCP genes in leaf tissue and fruits at different developmental stages were interrogated using hierarchical clustering. A heatmap of the expression patterns of the papaya PLCP genes is shown in Fig. 3.

Among the 33 PLCP genes identified in papaya genome, ten of them, *CpPAP2*, *CpPAP3*, *CpPAP4*, *CpPAP5*, *CpPAP10*, *CpCEP1*, *CpRD19C*, *CpXBCP1*, *CpXCP4*, and *CpXCP9*, exhibited a basal level of expression in leaf and fruits across all developmental stages. Five papaya PLCP genes, *CpRD21B*, *CpRD19A*, *CpRD19B*, *CpAALP*, and *CpCTB1*, showed constant expression in leaf and fruits across all developmental stages. The rest of the papaya PLCP genes exhibited either tissue-specific or developmental stage-specific expression patterns. In general, the papaya PLCP genes of subfamily VI showed a relatively lower level of expression compared to the rest of the PLCP subfamilies although papaya contains more PLCP genes in subfamily VI than the ones in other subfamilies.

We paid special attention to subfamily III because all the major PLCPs purified from papaya latex including papain, which is responsible for the defense of papaya against herbivorous insects [28], were grouped in this subfamily. Overall, PLCP genes of subfamily III expressed at relatively higher levels in leaf tissue and fruits at early developmental stages than the fruits at late developmental stages. Papain gene (*CpXCP5*) showed the highest expression in fruits at stage 1 and 30 DPA. The two closest paralogous genes of *CpXCP5*, *CpXCP4* and *CpXCP9*, exhibited a low level of expression in leaf and fruits at all developmental stages. *CpXCP3* and *CpXCP8*, a pair of paralogous genes derived from a recent duplication event, showed a high level of expression in leaf tissue and fruits at early developmental stages, while *CpXCP3* showed a much higher level of expression in fruit at 150 DPA than *CpXCP8*. *CpXCP6* and *CpXCP7*, another pair of paralogous PLCP genes derived from a recent duplication event, exhibited similar expression patterns in all tested tissues.

**Fig. 4:**
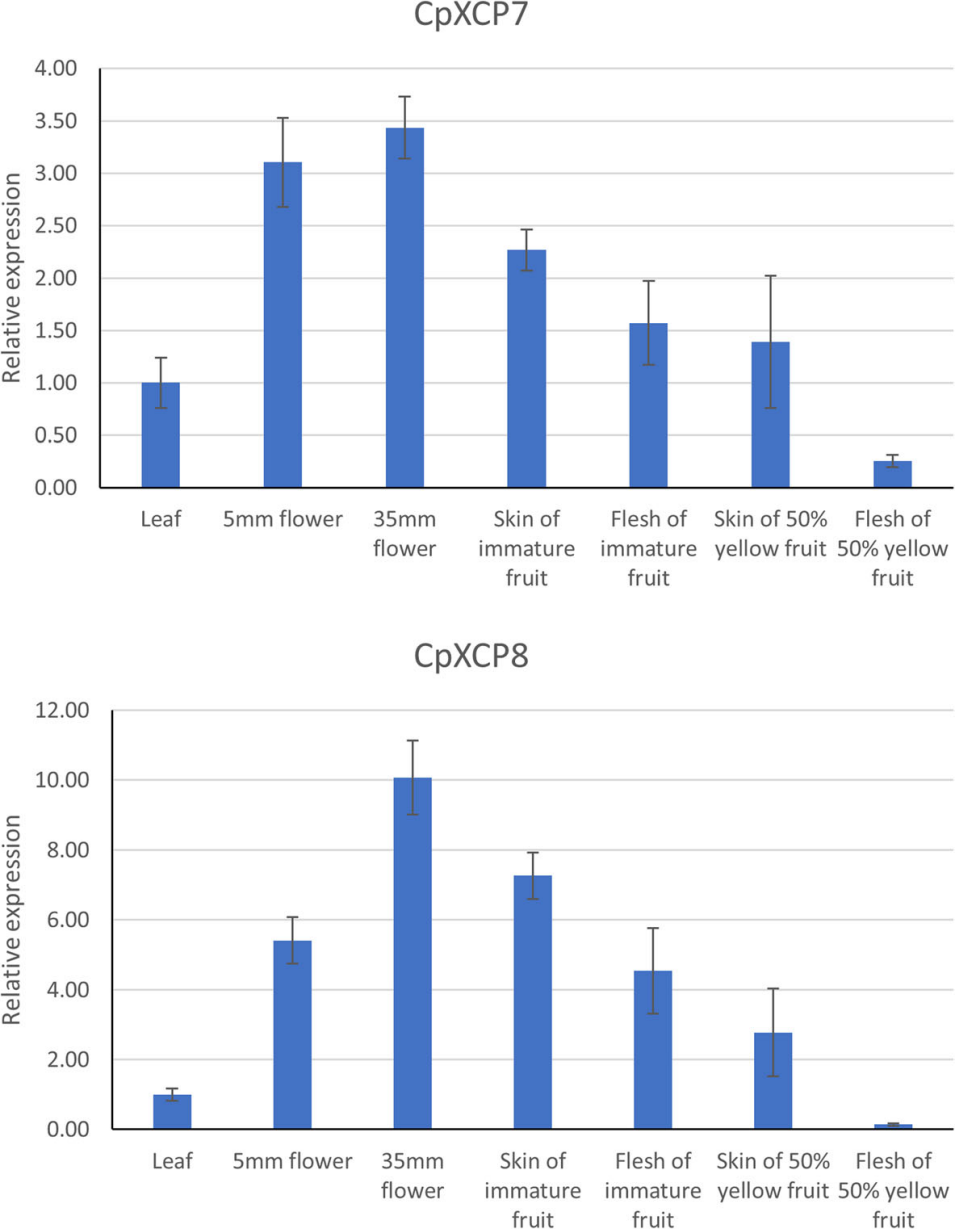
Quantitative Real-time PCR result of *CpXCP7* and *CpXCP8* across different tissues. Bar graph shows relative *CpXCP7* and *CpXCP8* gene expression in seven different tissues. Their expression in leaf tissue was chosen to be calibrator

Papaya latex is usually harvested from fruit skin of green papaya fruits by mechanical wounding. We selected *CpXCP7* and *CpXCP8* that encode glycyl endopeptidase and chymopapain, the two major PLCPs in papaya latex, and further examined their expression patterns in the skin of papaya fruit using quantitative real-time PCR. Our result showed *CpXCP7* and *CpXCP8* expressed at a higher level in the skin than that in the flesh of the papaya fruit (Fig. 4). As indicated by RNA-Seq result, *CpXCP7* and *CpXCP8* showed a higher level of expression in immature green fruit than the one in mature fruit. The highest expression was observed in mature male flower for both *CpXCP7* and *CpXCP8*. And both expressed at a relatively higher level in mature male flower than the one in male flower before meiosis stage.

### Lineage-specific expansions of PLCP genes are mostly derived from tandem duplication

Lineage-specific expansions of gene families may result from genome-wide duplication, as well as segmental and tandem duplication events. To examine whether lineage-specific expansions of PLCP genes are driven by genome-wide duplication or tandem duplication events, we carefully looked at the chromosome locations of PLCP genes for two monocot (*O. sativa* and *S. bicolor*) and three dicot species (*A. thaliana*, *V. vinifera*, and *P. trichocarpa*). We also used MCScanX to classify the gene duplication events that led to lineage-specific expansions of PLCP genes in the seven selected plant species.

**Fig. 5:**
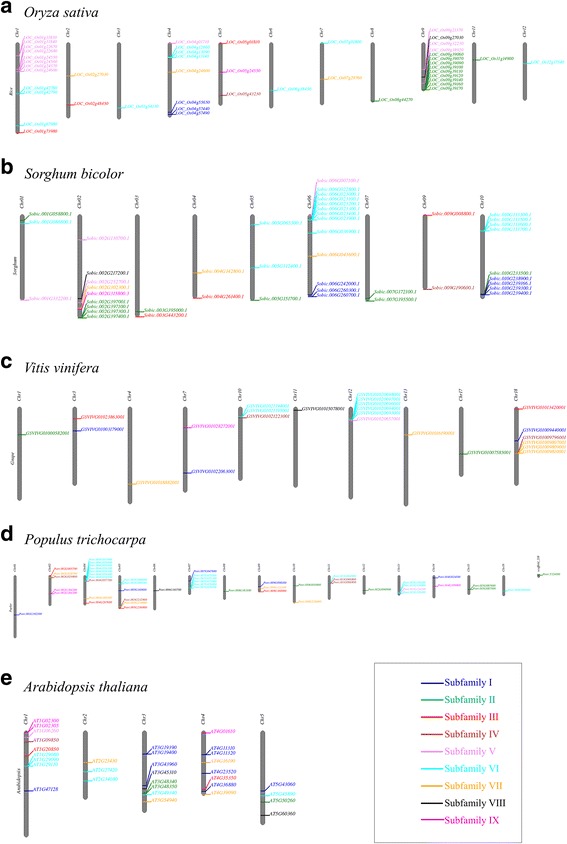
Chromosomal distribution of PLCP genes in rice (**a**), sorghum (**b**), grape (**c**), poplar (**d**) and *Arabidopsis* (**e**). The PLCP genes were color-coded according to their classification in PLCP subfamilies

We mapped PLCP genes of the five plant species on their chromosomes (Fig. 5). Our result clearly displayed the uneven distribution of PLCPs along the chromosomes. PLCP genes from the same subfamilies formed clusters on chromosomes (Fig. 5). Subfamily VI contains the largest number of PLCP genes. Clusters of subfamily VI PLCP genes were observed in all five plant species. Our phylogenetic analysis revealed lineage-specific gene expansion of subfamilies II and V PLCP genes in rice. Consisting with this, a cluster of subfamily II PLCP genes and a cluster of subfamily V PLCP genes were observed on rice chromosome 9 and chromosome 1, respectively. Similarly, PLCP genes of subfamilies I, II, and VI formed clusters on sorghum chromosomes 10, 2, and 6, respectively. We further used MCScanX to exam the origins of the gene duplication events that led to lineage-specific expansions of PLCP genes in the seven selected plant species. Additional file 9 showed the percentages of PLCP genes derived from tandem duplication events in each PLCP subfamily. As we have described above, our MCScanX result also strongly supported lineage-specific expansions of PLCP genes were mostly derived from tandem duplications, not from genome-wide duplications.

## Discussion

Gene families are groups of genes that share important characteristics, derived from gene duplication events followed by mutation and divergence. Duplication events can occur through frequent tandem duplications, or infrequent large-scale segmental duplications or whole-genome duplications. Gene duplication provides raw genetic material for natural selection which resulted in adaptive evolution, novel traits and speciation [49, 50]. Therefore, studying the evolutionary pathways that led to the emergence of novel functions can greatly enhance our understanding of plant adaptations.

PLCPs are proteolytic enzymes that are involved in a broad range of biological processes such as senescence, programmed cell death, pollen development, fruit ripening, and seed germination [13]. In this study, we identified PLCP genes in seven plant species. Significant variations in size of the gene family were observed among species and among different PLCP subfamilies, which may reflect genetic adaptation to different environments in different plant species. We grouped the PLCPs into 9 subfamilies. Based on the protein structures, subfamilies I-VI contain cathepsin L-like PLCPs, and subfamilies VII, VIII and IX contain cathepesin F-like, cathepsin H-like and cathepsin B-like PLCPs, respectively. Interestingly, subfamilies VII, VIII and IX showed no variation or only slight variations in size among species. In contrast, subfamilies I-VI exhibited striking variations in size among species. Studies showed that dosage-sensitive genes normally exhibit less expression variation among tissues [51]. Consistently, no significant expression variation was observed for papaya PLCP genes of subfamilies VII, VIII and IX in leaf and fruits across all developmental stages. In contrast, papaya PLCP genes of subfamilies I-VI mostly exhibited either tissue-specific or developmental stage-specific expression patterns. It might be interesting to test whether PLCP genes of subfamilies VII, VIII and IX are dosage-sensitive genes in the future study.

Lineage-specific expansion of PLCPs was observed in subfamilies I-VI. Variations in size of gene family among species may result from genome-wide, large-scale segmental or tandem duplications followed by differential retentions. Our result revealed that tandem duplications played the dominant role in affecting copy numbers of PLCPs in plants. Cannon et al. studied gene duplications for 50 large gene families in *Arabidopsis thaliana* and found that highly conserved, housekeeping or key regulatory gene families were over-represented in the class of gene families with low tandem duplications, while gene families involving pathogen defense or diverse enzymatic functions were over-represented in the class of gene families with medium and high tandem duplications [52]. By studying duplicated genes in four land plants, Hanada et al. demonstrated that genes expanded via tandem duplication tend to be involved in responses to environmental stimuli, while genes expanded via non-tandem duplication mechanisms tend to be involved in primary metabolic and cellular functions [53]. All together suggested that PLCPs of subfamilies I-VI with dynamic variations might be associated with evolutionary adaptive traits. However, we can’t exclude the possibility that some of these expansions have no adaptive significance.

Approximately 10% of all angiosperm plant species exude latex upon damage [54] and papaya is one of them. Papain, one of the PLCPs in latex of papaya, plays the key role in protecting papaya from herbivorous insects [28]. Papaya showed clear lineage-specific gene expansion in the subfamily III of PLCP genes. Interestingly, all the four major PLCPs purified from papaya latex, including papain, chymopapain, glycyl endopeptidase and caricain, were grouped into the lineage-specific expansion branch in the subfamily III. Latex is a highly convergent trait that has evolved independently multiple times in plants. Since laticifers, the specialized cells that synthesize and accumulate latex, are absence in primitive angiosperms, the latex trait likely evolved recently [55]. Articulated laticifers are found in all Caricaceae species, but absent in its closest sister family Moringaceae [56], suggesting the latex trait in Caricaceae evolved after Caricaceae and Moringaceae diverged from a common ancestor approximately 65 MYA [57]. Our result is consistent with this prediction that all the major PLCPs of papaya latex evolved recently.

We found majority of the dicot species contain two PLCP genes of subfamily III, while papaya contains at least nine PLCP genes of subfamily III. PLCPs of subfamily III are the major components of papaya latex and play important role in protecting papaya against herbivore attack. According to the widely accepted ‘plant-herbivore coevolution’ theory, plant and its feeding insects have engaged in an evolutionary antagonistic interaction that led to the repeated diversification of plant defense strategies to avoid extinction [58, 59]. Therefore, coevolution might be central to understanding the causes of lineage-specific expansion and diversification of subfamily III PLCPs in papaya.

The family Caricaceae consists of six genera and 35 species [60]. It originated in Africa approximately 65 MYA and dispersed from Africa to Central America approximately 35 MYA [60]. In the New World, the Neotropical Caricaceae migrated further southward through Central American bridge to South America approximately 27 MYA. Our phylogenetic analysis revealed that papaya PLCPs of subfamily III expanded via stepwise duplication events. Since the PLCP genes of subfamily III from *Vasconcellea* species were also included in our phylogenetic analysis, our phylogenetic tree indicated that the lineage-specific gene expansion of subfamily III initiated before *Carica* and *Vasconcellea* diverged from a common ancestor and had undergone additional expansion after these two genera split from a common ancestor about 27 MYA. Furthermore, the estimated ages of the lineage-specific gene expansion events of subfamily III PLCPs in Caricaceae were coincident with the dispersal of Caricaceae from Africa to Central America and further dispersal from Central America to South America. The plant-herbivore coevolutionary theory proposed that the continuous reciprocal selective effects of herbivore attack and plant defense shaped patterns of divergence among related species [59]. The diversity of defense-related compounds in plants is largely promoted by insect detoxification mechanisms [59]. During the migration, Caricaceae might have had unprecedented interactions with herbivore species which it had never encountered before. Therefore, new and stronger cysteine proteases could have possibly evolved in response to the changing herbivore attack. The lineage-specific expansion of papaya PLCPs of subfamily III might result from the continuous reciprocal selective effects of herbivore attack and plant defense.

## Conclusions

Tandem duplications played the dominant role in affecting copy number of PLCP genes in plants. Significant variations in size of the PLCP subfamilies among species may reflect genetic adaptation of plant species to different environments. The lineage-specific expansion of papaya PLCPs of subfamily III might have been promoted by the continuous reciprocal selective effects of herbivore attack and plant defense.

## Additional files

Additional file 1: Table S1. Details of papaya tissues used for quantitative real-time PCR analysis. (XLSX 11 kb)

Additional file 2: Table S2. Details of papaya tissues used for RNA-Seq library construction. (XLSX 12 kb)

Additional file 3: Table S3. Primers used for quantitative real-time PCR. (XLSX 9 kb)

Additional file 4: Table S4. List of species used for construction of the phylogenetic tree of subfamily III PLCP genes. (XLSX 12 kb)

Additional file 5: Table S5. List of the 155 PLCP genes of subfamily III used for phylogenetic analysis. (XLSX 16 kb)

Additional file 6: Table S6. Estimates of synonymous and non-synonymous nucleotide divergence of subfamily III PLCP gene pairs in papaya. (DOCX 19 kb)

Additional file 7: Table S7. Estimated age of divergence of subfamily III PLCP gene pairs in papaya. (DOCX 13 kb)

Additional file 8: Figure S1. Estimated ages of divergence time (middle panel) and silent-site divergence (bottom panel) of subfamily III PLCP gene pairs in papaya. The corresponding node of the estimated divergence time for each group is shown on the top panel. (TIFF 3175 kb)

Additional file 9: Table S8. The percentages of PLCP genes derived from tandem duplication events in each PLCP subfamily estimated by MCScanX. (XLSX 12 kb)

## Abbreviations

ANOVA: Analysis of variance
bp: base pair
CDD: Conserved domains database
DPA: Days post anthesis
FPKM: Fragments per kilobase of exon per million fragments mapped
Ma: Megaannus
MYA: Million years ago
PCD: Programmed cell death
PLCPs: Papain-like cysteine proteases
qPCR: Quantitative real-time PCR
