# Supplementary material for: Papain-like cysteine proteases in Carica papaya: lineage-specific gene duplication and expansion
Source: BMC Genomics. 2018 Jan 6;19:26. doi: 10.1186/s12864-017-4394-y (PMC5756445; doi:10.1186/s12864-017-4394-y)
Supplement: Supplementary file 6 — Estimates of synonymous and non-synonymous nucleotide divergence of subfamily III PLCP gene pairs in papaya. (DOCX 19 kb) [file 12864_2017_4394_MOESM6_ESM.docx]

**Additional file 6: Table S6** Estimates of synonymous and non-synonymous nucleotide divergence of subfamily III PLCP gene pairs in papaya.

| **Gene pair** | |  | **No. sites (bp)** | | | | **Sequence divergence** | |  |
| --- | --- | --- | --- | --- | --- | --- | --- | --- | --- |
|  |  |  | **Total sites** | **Total coding sites** | **Syn.**  **sites** | **Non-syn.**  **sites** | **Syn. sites (*Ks*)** | **Non-syn. sites (*Ka*)** | ***Ka*/*Ks*** |
| CpXCP4 | CpXCP9 |  | 1540 | 1005 | 234.83 | 770.17 | 0.00427 | 0.0026 | 0.608899 |
| CpXCP3 | CpXCP8 |  | 1468 | 1080 | 239.33 | 843.67 | 0.01264 | 0.01315 | 1.040348 |
| CpXCP6 | CpXCP7 |  | 1471 | 1044 | 235.17 | 808.83 | 0 | 0.00124 | N/A |
| CpXCP4 | CpXCP5 |  | 1381 | 879 | 206.75 | 672.25 | 0.2605 | 0.08081 | 0.310211 |
| CpXCP5 | CpXCP9 |  | 1381 | 879 | 206.75 | 672.25 | 0.26738 | 0.08081 | 0.302229 |
| CpXCP5 | CpXCP6 |  | 1287 | 861 | 197.75 | 663.25 | 0.35696 | 0.18518 | 0.51877 |
| CpXCP5 | CpXCP7 |  | 1296 | 861 | 197.75 | 663.25 | 0.35696 | 0.18325 | 0.513363 |
| CpXCP4 | CpXCP7 |  | 1446 | 1014 | 232.25 | 781.75 | 0.41295 | 0.20337 | 0.492481 |
| CpXCP4 | CpXCP6 |  | 1438 | 1017 | 232.58 | 784.42 | 0.41216 | 0.20424 | 0.495536 |
| CpXCP6 | CpXCP9 |  | 1436 | 1011 | 231.58 | 779.42 | 0.42208 | 0.20238 | 0.479483 |
| CpXCP7 | CpXCP9 |  | 1445 | 1011 | 231.58 | 779.42 | 0.33 | 0.20 | 0.6047 |
| CpXCP8 | CpXCP9 |  | 1400 | 888 | 202.67 | 685.33 | 0.48845 | 0.32387 | 0.663057 |
| CpXCP5 | CpXCP8 |  | 1253 | 867 | 195.08 | 671.92 | 0.56668 | 0.27657 | 0.488053 |
| CpXCP3 | CpXCP5 |  | 1260 | 885 | 192.83 | 692.17 | 0.50045 | 0.2637 | 0.526926 |
| CpXCP4 | CpXCP8 |  | 1401 | 1014 | 229.25 | 784.75 | 0.61045 | 0.2724 | 0.446228 |
| CpXCP3 | CpXCP9 |  | 1402 | 1014 | 229.58 | 784.42 | 0.62729 | 0.26373 | 0.420428 |
| CpXCP6 | CpXCP8 |  | 1399 | 1038 | 231.25 | 806.75 | 0.68116 | 0.2145 | 0.314904 |
| CpXCP7 | CpXCP8 |  | 1399 | 1041 | 231.92 | 809.08 | 0.68154 | 0.21983 | 0.322549 |
| CpXCP3 | CpXCP6 |  | 1399 | 1041 | 231.25 | 809.9275 | 0.6956 | 0.21797 | 0.313355 |
| CpXCP3 | CpXCP4 |  | 1415 | 1017 | 230.25 | 610.25 | 0.61454 | 0.26641 | 0.433511 |
| CpXCP3 | CpXCP7 |  | 1404 | 1041 | 231.25 | 809.75 | 0.6956 | 0.21962 | 0.315727 |

syn., synonymous; non-syn., non-synonymous.
