# Supplementary material for: Papain-like cysteine proteases in Carica papaya: lineage-specific gene duplication and expansion
Source: BMC Genomics. 2018 Jan 6;19:26. doi: 10.1186/s12864-017-4394-y (PMC5756445; doi:10.1186/s12864-017-4394-y)
Supplement: Supplementary file 7 — Estimated age of divergence of subfamily III PLCP gene pairs in papaya. (DOCX 13 kb) [file 12864_2017_4394_MOESM7_ESM.docx]

**Additional file 7: Table S7** Estimated age of divergence of subfamily III PLCP gene pairs in papaya.

| **Gene pair** | | **Silent site divergence (*Ksil*)** | **Estimated age (MYA)** |
| --- | --- | --- | --- |
|  |  |  |  |
| CpXCP4 | CpXCP9 | 0.00131 | 0.137 |
| CpXCP3 | CpXCP8 | 0.0113 | 1.185 |
| CpXCP6 | CpXCP7 | 0 | N/A |
| CpXCP4 | CpXCP5 | 0.15767 | 16.523 |
| CpXCP5 | CpXCP9 | 0.1594 | 16.704 |
| CpXCP5 | CpXCP6 | 0.3102 | 32.508 |
| CpXCP5 | CpXCP7 | 0.312 | 32.696 |
| CpXCP4 | CpXCP7 | 0.32909 | 34.499 |
| CpXCP4 | CpXCP6 | 0.3292 | 34.499 |
| CpXCP6 | CpXCP9 | 0.3305 | 34.635 |
| CpXCP7 | CpXCP9 | 0.3319 | 34.782 |
| CpXCP8 | CpXCP9 | 0.3341 | 35.012 |
| CpXCP5 | CpXCP8 | 0.3856 | 40.409 |
| CpXCP3 | CpXCP5 | 0.4024 | 42.170 |
| CpXCP4 | CpXCP8 | 0.403 | 42.233 |
| CpXCP3 | CpXCP9 | 0.4241 | 44.444 |
| CpXCP6 | CpXCP8 | 0.4462 | 46.760 |
| CpXCP7 | CpXCP8 | 0.4476 | 46.906 |
| CpXCP3 | CpXCP6 | 0.4577 | 47.965 |
| CpXCP3 | CpXCP4 | 0.45925 | 48.127 |
| CpXCP3 | CpXCP7 | 0.4648 | 48.709 |
