# Supplementary figures and images for: Papain-like cysteine proteases in Carica papaya: lineage-specific gene duplication and expansion
Source: BMC Genomics. 2018 Jan 6;19:26. doi: 10.1186/s12864-017-4394-y (PMC5756445; doi:10.1186/s12864-017-4394-y)

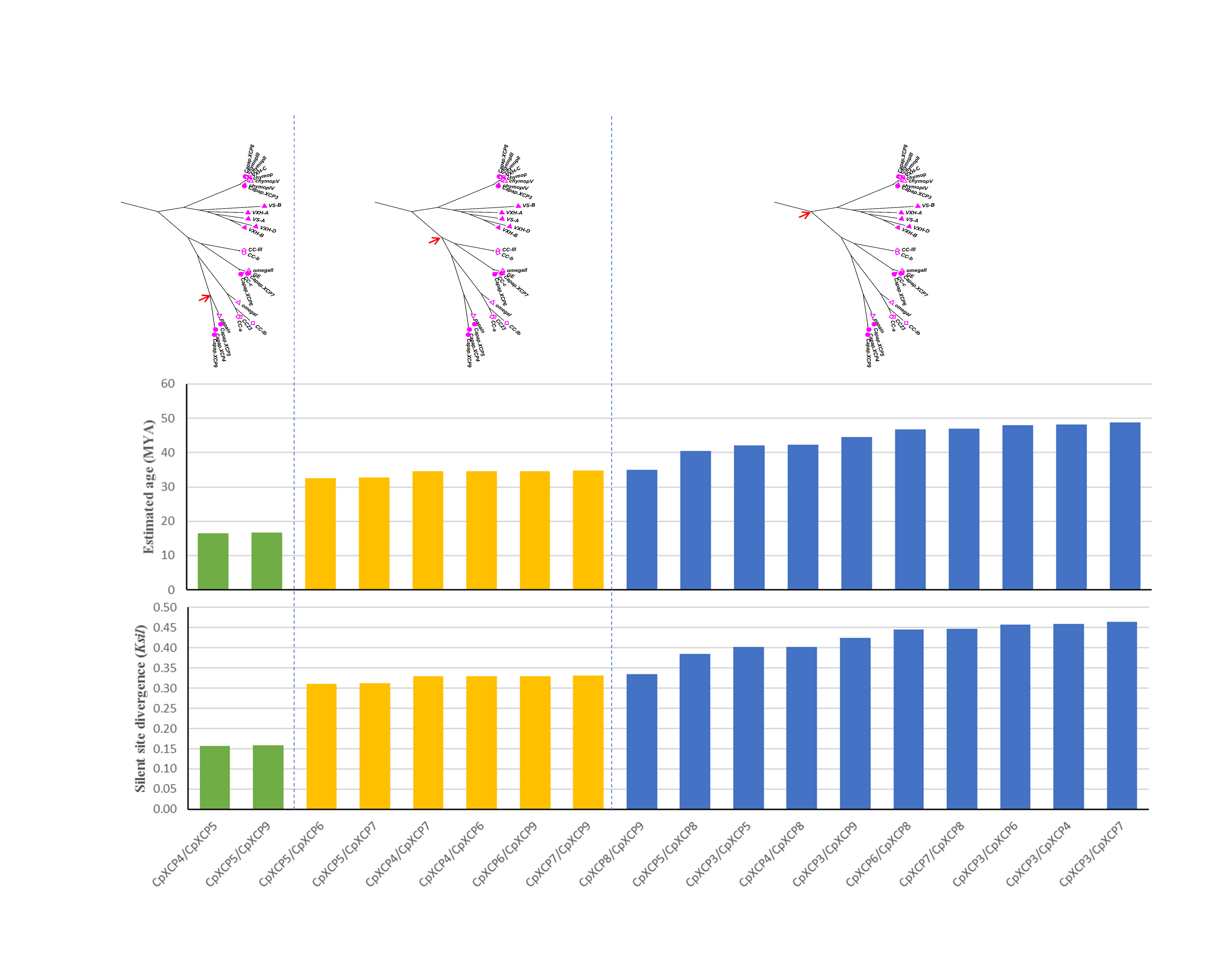

Supplement: Supplementary file 8 — Estimated ages of divergence time (middle panel) and silent-site divergence (bottom panel) of subfamily III PLCP gene pairs in papaya. The corresponding node of the estimated divergence time for each group is shown on the top panel. (TIFF 3175 kb) [file 12864_2017_4394_MOESM8_ESM.tif]
